# Rapid normal fibroblast‐tumor crosstalk promotes cancer‐associated fibroblast‐like activation and attenuates mitomycin C cytotoxicity in bladder cancer

**DOI:** 10.1002/ccs3.70110

**Published:** 2026-08-24

**Authors:** Jinhui Gao, Cindy Xinyu Ji, Ci Ren, John Hines, Eleanor Stride, Richard T. Bryan, Jennifer L. Rohn

**Affiliations:** ^1^ Division of Medicine University College London London UK; ^2^ Cancer Research UK Manchester Institute University of Manchester Manchester UK; ^3^ Westmoreland Street University College London Hospital London UK; ^4^ Department of Engineering Science Institute of Biomedical Engineering University of Oxford Oxford UK; ^5^ Department of Cancer and Genomic Sciences, Bladder Cancer Research Centre, College of Medicine and Health University of Birmingham Birmingham UK

**Keywords:** bladder cancer, cancer‐associated fibroblast, EMT, mitomycin C, normal fibroblast

## Abstract

Normal fibroblasts (NFs) are among the earliest stromal cells encountered by bladder cancer cells during invasion into the lamina propria, yet their contribution to tumor progression and intravesical chemotherapy response remains poorly defined. Here, we investigated whether NFs are passive stromal bystanders or active participants in early bladder cancer microenvironmental remodeling. Analysis of an early‐stage non‐muscle‐invasive bladder cancer transcriptomic cohort revealed that fibroblast‐associated scores were higher in T1 than Ta tumors, enriched in European Organisation for Research and Treatment of Cancer high‐risk disease, positively correlated with epithelial‐mesenchymal transition (EMT) scores, and associated with increased expression of resistance‐linked genes. Experimentally, fibroblast‐conditioned medium reduced bladder cancer cell proliferation while accelerating wound closure, indicating a less proliferative but more motile phenotype. This was accompanied by EMT‐like cadherin remodeling, including reduced E‐cadherin and increased N‐cadherin staining. Reciprocally, tumor‐derived signals rapidly induced cancer‐associated fibroblast (CAF)‐like activation features in NFs within 48 h, including increased α‐smooth muscle actin, fibroblast activation protein, and PDGFRβ expression. Functionally, increasing fibroblast‐to‐tumor cell ratios progressively attenuated mitomycin C (MMC)‐induced cytotoxicity, demonstrating that stromal context can modify chemotherapy response in vitro. Together, these findings show that NF‐tumor crosstalk is rapid, bidirectional and functionally relevant to MMC response in vitro. Rather than acting only after stable CAF formation, NFs can rapidly acquire CAF‐like activation features following tumor‐derived stimulation and contribute to a stromal context associated with EMT‐like plasticity and reduced MMC‐induced cytotoxicity in vitro.

## Graphical abstract

Normal bladder fibroblasts rapidly engage in bidirectional crosstalk with bladder cancer cells, acquiring cancer‐associated fibroblast‐like activation features while promoting wound closure, epithelial‐mesenchymal transition‐like cadherin remodeling, and reduced mitomycin C‐induced cytotoxicity. These findings identify stromal context as an early modifier of tumor behavior and chemotherapy response.

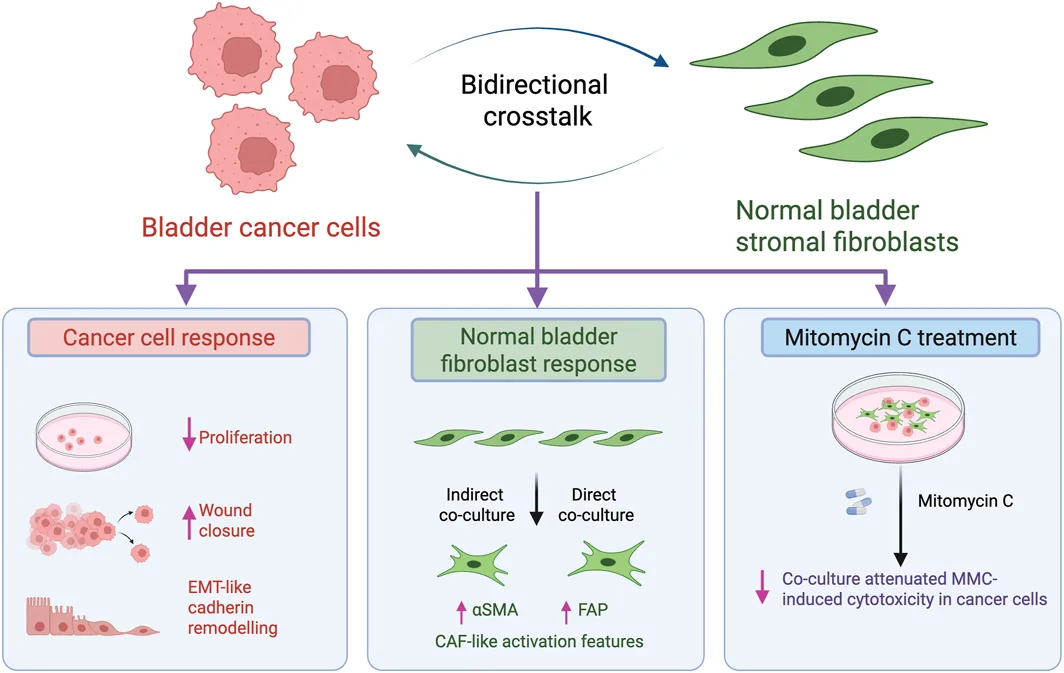

## 1 INTRODUCTION

Bladder cancer is among the most common malignancies worldwide, with non‐muscle‐invasive disease (NMIBC) accounting for 75% of diagnoses.^1^ Despite standard‐of‐care transurethral resection, BCG immunotherapy, and intravesical mitomycin C (MMC), recurrence rates reach 50%–80%, and 10%–20% of cases progress to muscle‐invasive disease.^2^, ^3^ MMC remains widely used, particularly in the perioperative and adjuvant NMIBC setting, but treatment responses are variable.^4^ This variability is usually interpreted through tumor‐intrinsic features such as grade, stage, and molecular subtype; however, the local stromal environment encountered by tumor cells may also influence both disease behavior and therapeutic response.^5^

The bladder lamina propria represents a critical anatomical interface in early disease progression.^6^ As urothelial tumor cells breach the basement membrane and invade from Ta into T1 disease, they encounter a connective tissue compartment rich in extracellular matrix, vascular structures, immune cells, and resident fibroblasts.^7^ Normal fibroblasts (NFs) are therefore not distant or late participants in bladder cancer progression; they are positioned at one of the earliest stromal contact points along the invasion axis.^8^ This anatomical position is particularly significant: as bladder cancer progresses from the urothelium (NMIBC) through the lamina propria toward the detrusor muscle (MIBC), invading tumor cells are likely to encounter NFs among the earliest stromal populations.^9^ Despite this, most bladder cancer studies have focused on established cancer‐associated fibroblasts (CAFs), while the role of their NF precursors remains comparatively underexplored. This distinction is important because CAF‐rich tumors do not arise de novo. They are likely generated through dynamic tumor‐stromal interactions in which NFs progressively acquire altered activation states in response to tumor‐derived signals.

CAFs are established regulators of tumor invasion, extracellular matrix remodeling, immune suppression, and therapy resistance across multiple cancers.^10^, ^11^ In bladder cancer, fibroblast‐rich and stromal‐enriched tumor states have been associated with aggressive behavior and poorer therapeutic responses. However, a key unresolved question is whether NFs can actively shape bladder cancer phenotypes before becoming fully established CAFs. If NFs can rapidly alter tumor behavior, and if tumor cells can reciprocally activate fibroblasts within clinically relevant timeframes, then early tumor‐stromal crosstalk may represent a previously underappreciated determinant of NMIBC progression and intravesical chemotherapy failure.

Studies in lung and colorectal cancer demonstrate that NFs actively remodel cancer phenotypes through paracrine signals and ECM interactions,^12^, ^13^ but equivalent bidirectional interactions remain uncharacterized in bladder cancer. Two processes are particularly underexplored. First, fibroblast‐derived paracrine signals may promote epithelial‐to‐mesenchymal transition‐like plasticity in bladder cancer cells, shifting tumor cells toward a more migratory phenotype. Second, tumor cells may rapidly reprogram NFs toward CAF‐like states, cytoskeletal remodeling and altered secretory behavior. These bidirectional changes could have direct functional consequences for chemotherapy response, particularly for MMC, whose activity depends on tumor cell exposure during a limited intravesical treatment window.^14^

We hypothesized that NFs actively participate in shaping bladder cancer phenotype and treatment response. We combined analysis of an early‐stage NMIBC transcriptomic cohort with indirect and direct co‐culture models of bladder cancer cells and NFs. We first assessed whether fibroblast‐associated transcriptional signals are linked to stage, risk group, epithelial‐mesenchymal transition (EMT)‐associated features and progression‐free survival (PFS) patterns in patient tumors. We then experimentally examined whether fibroblast‐derived soluble factors alter tumor proliferation, migration, and EMT‐like marker expression, whether bladder cancer cells reciprocally activate NFs toward CAF‐like states, and whether this interaction modifies MMC sensitivity. Together, these data define rapid NF‐tumor crosstalk as a bidirectional and therapeutically relevant process in bladder cancer.

## 2 RESULTS

### 2.1 Fibroblast‐associated transcriptional programs are enriched in high‐risk NMIBC

**FIGURE 1:**
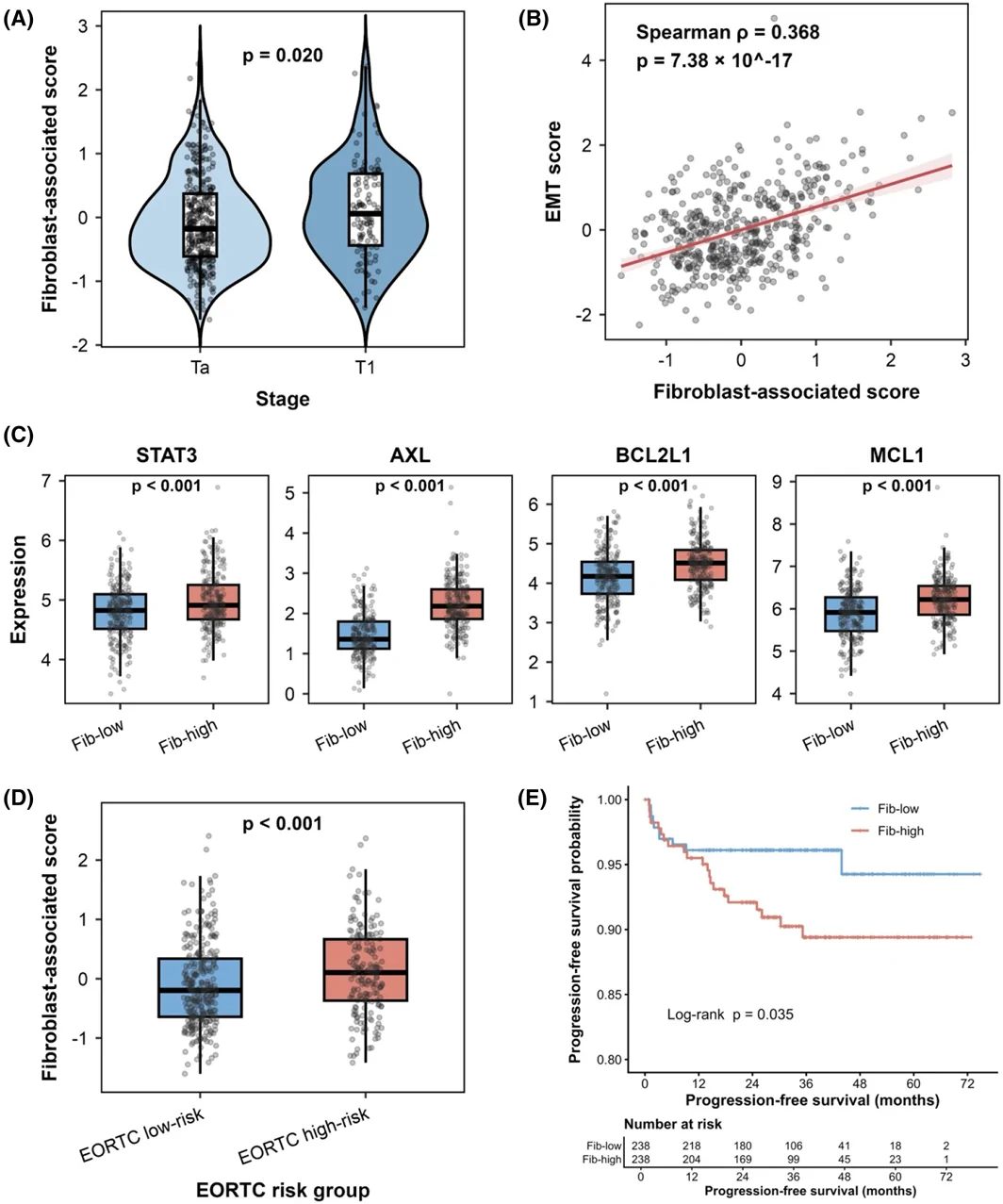
Fibroblast‐associated transcriptional programs are linked to invasive stage, EMT features, risk stratification, and PFS in NMIBC tumors. (A) Violin and box plots showing the distribution of fibroblast‐associated scores in Ta versus T1 stage NMIBC tumors. (B) Scatter plot demonstrating a significant positive correlation between fibroblast‐associated scores and EMT scores across the transcriptomic cohort. The red line indicates the linear regression fit, with the shaded area representing the 95% confidence interval. (C) Box plots illustrating the mRNA expression levels of pro‐survival signaling, EMT plasticity, and cytotoxic stress resistance genes (STAT3, AXL, BCL2L1, and MCL1) stratified by fibroblast‐low (Fib‐low) and fibroblast‐high (Fib‐high) tumor groups. (D) Box plot comparing fibroblast‐associated scores between EORTC low‐risk and EORTC high‐risk clinical groups. (E) Kaplan–Meier survival curve showing PFS probability over time (months) for patients with Fib‐low (blue line) versus Fib‐high (red line) tumors. The corresponding number‐at‐risk table is provided below the axis. EMT, epithelial‐mesenchymal transition; EORTC, European Organisation for Research and Treatment of Cancer; NMIBC, non‐muscle‐invasive bladder cancer; PFS, progression‐free survival.

To determine whether fibroblast‐associated biology is already evident in early‐stage bladder cancer, we analyzed an NMIBC transcriptomic cohort containing annotated clinicopathological and PFS data (*n* = 476 tumors; Ta, *n* = 345; T1, *n* = 112). A fibroblast‐associated score was generated from a panel of fibroblast and stromal activation genes and compared across disease stage and risk groups. Fibroblast‐associated scores were significantly higher in T1 tumors compared with Ta tumors, indicating that fibroblast‐related transcriptional programs increase with lamina propria invasion. This observation supports the concept that fibroblast involvement is not restricted to late‐stage disease but is already detectable at the transition from noninvasive to invasive NMIBC (Figure 1A).

We next asked whether fibroblast‐associated transcriptional enrichment was linked to EMT‐like tumor biology. Fibroblast‐associated scores positively correlated with EMT scores across the cohort, suggesting that tumors with stronger fibroblast‐associated signals also display greater mesenchymal transcriptional features (Figure 1B).

Because stromal‐rich microenvironments can support treatment resistance, we compared resistance‐linked and pro‐survival genes between fibroblast‐low and fibroblast‐high tumors. Fibroblast‐high tumors showed increased expression of STAT3, AXL, BCL2L1, and MCL1, genes associated with pro‐survival signaling, EMT‐associated plasticity, and resistance to cytotoxic stress (Figure 1C). Consistent with this, European Organisation for Research and Treatment of Cancer (EORTC) high‐risk tumors showed significantly higher fibroblast‐associated scores than EORTC low‐risk tumors (low‐risk, *n* = 286; high‐risk, *n* = 174). Sample numbers varied between analyses because not all tumors had complete stage, EORTC‐risk, and survival annotation. These findings indicate that fibroblast‐associated biology is connected not only to anatomical invasion but also to clinically defined NMIBC risk (Figure 1D). Finally, Kaplan–Meier analysis showed lower PFS in fibroblast‐high than fibroblast‐low tumors (Figure 1E). To further assess whether the predefined fibroblast/stromal‐associated score was concordant with an independently published fibroblast marker set, we compared it with the MCP‐counter fibroblast transcriptomic markers^15^ in the same UROMOL NMIBC cohort. The predefined score showed a strong positive correlation with a mean *z*‐score derived from the published MCP‐counter fibroblast marker set (Spearman *ρ* = 0.897, *p* < 2.2e‐16, *n* = 476), with a similarly strong association observed using the restricted MCP‐counter fibroblast marker set (*ρ* = 0.900, *p* < 2.2e‐16, *n* = 476). Correlations for both the full and restricted MCP‐counter fibroblast marker sets are shown in Supporting Information S1: Figure S5.

### 2.2 NF‐ and tumor‐derived signals reciprocally suppress cell proliferation

**FIGURE 2:**
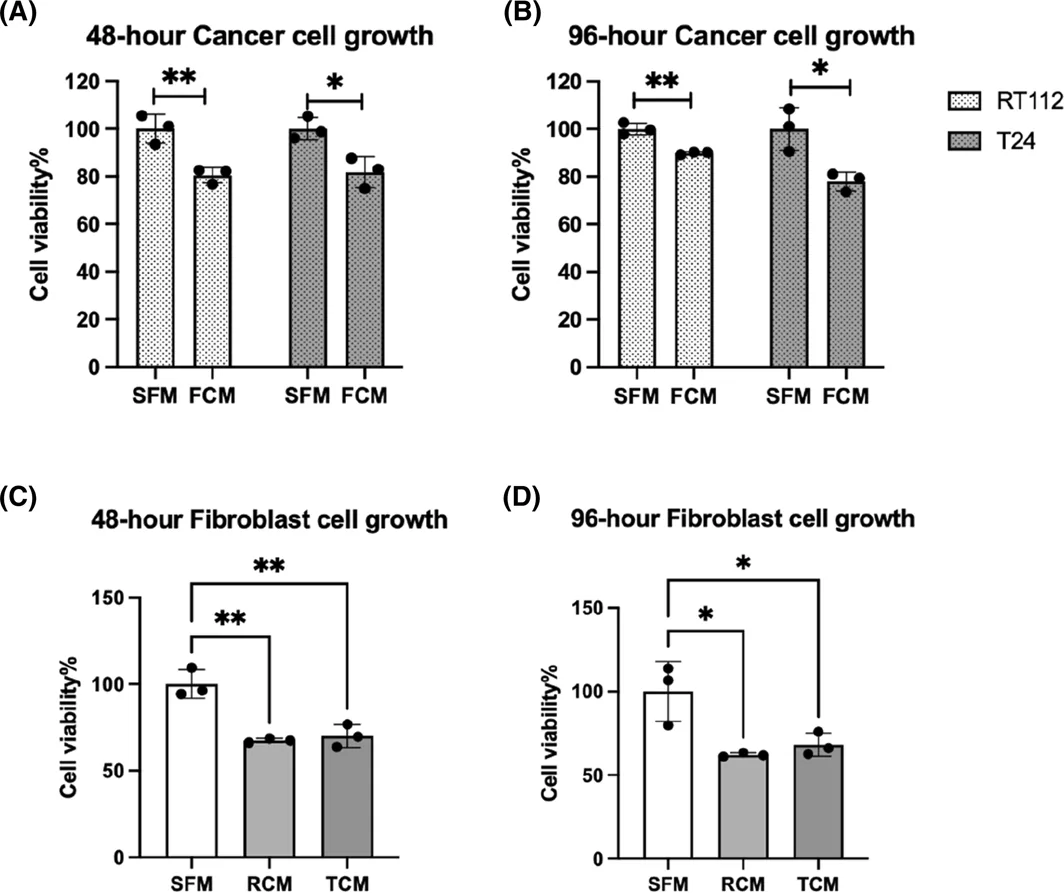
Reciprocal suppression of cell proliferation by tumor‐ and fibroblast‐derived conditioned media. (A, B) Proliferation of RT112 (low‐grade) and T24 (high‐grade) bladder cancer cell lines at 48 and 96 h following treatment with FCM or SFM, assessed by MTS assay. (C, D) Proliferation of NFs following exposure to RCM or TCM, compared with SFM. Data are mean ± SD; *n* = 3. *p ≤ 0.05; **p ≤ 0.01. FCM, fibroblast‐conditioned media; MTS, 3‐(4,5‐dimethylthiazol‐2‐yl)‐5‐(3‐carboxymethoxyphenyl)‐2‐(4‐sulfophenyl)‐2H‐tetrazolium; NFs, normal fibroblasts; RCM, RT112‐conditioned media; SFM, serum‐free media; TCM, T24‐conditioned media.

To explore the bidirectional effects between cancer cells and NFs, we first assessed how fibroblast conditioned media (FCM) influenced cancer cell proliferation, and subsequently assessed how cancer cell‐conditioned media affected NF proliferation. For the former, 3‐(4,5‐dimethylthiazol‐2‐yl)‐5‐(3‐carboxymethoxyphenyl)‐2‐(4‐sulfophenyl)‐2H‐tetrazolium (MTS) assays were conducted on RT112 and T24 bladder cancer cell lines. FCM treatment significantly reduced the proliferation of RT112 and T24 cells at 48 and 96 h (Figure 2A,B). Relative to serum‐free media (SFM) controls, FCM reduced RT112 proliferation to 80.5% at 48 h and 89.8% at 96 h, while T24 proliferation was reduced to 81.8% and 78.0%, respectively. The reciprocal interaction was equally pronounced: when NFs were treated with RT112‐conditioned media (RCM) or T24‐conditioned media (TCM), proliferation was significantly suppressed at 48 and 96 h. RCM reduced proliferation to 67.5% at 48 h and 62.1% at 96 h, while TCM led to reductions of 70.0% and 68.2%, respectively, relative to untreated controls (Figure 2C,D).

### 2.3 FCM reduces proliferation while enhancing wound closure and inducing EMT‐like cadherin remodeling

**FIGURE 3:**
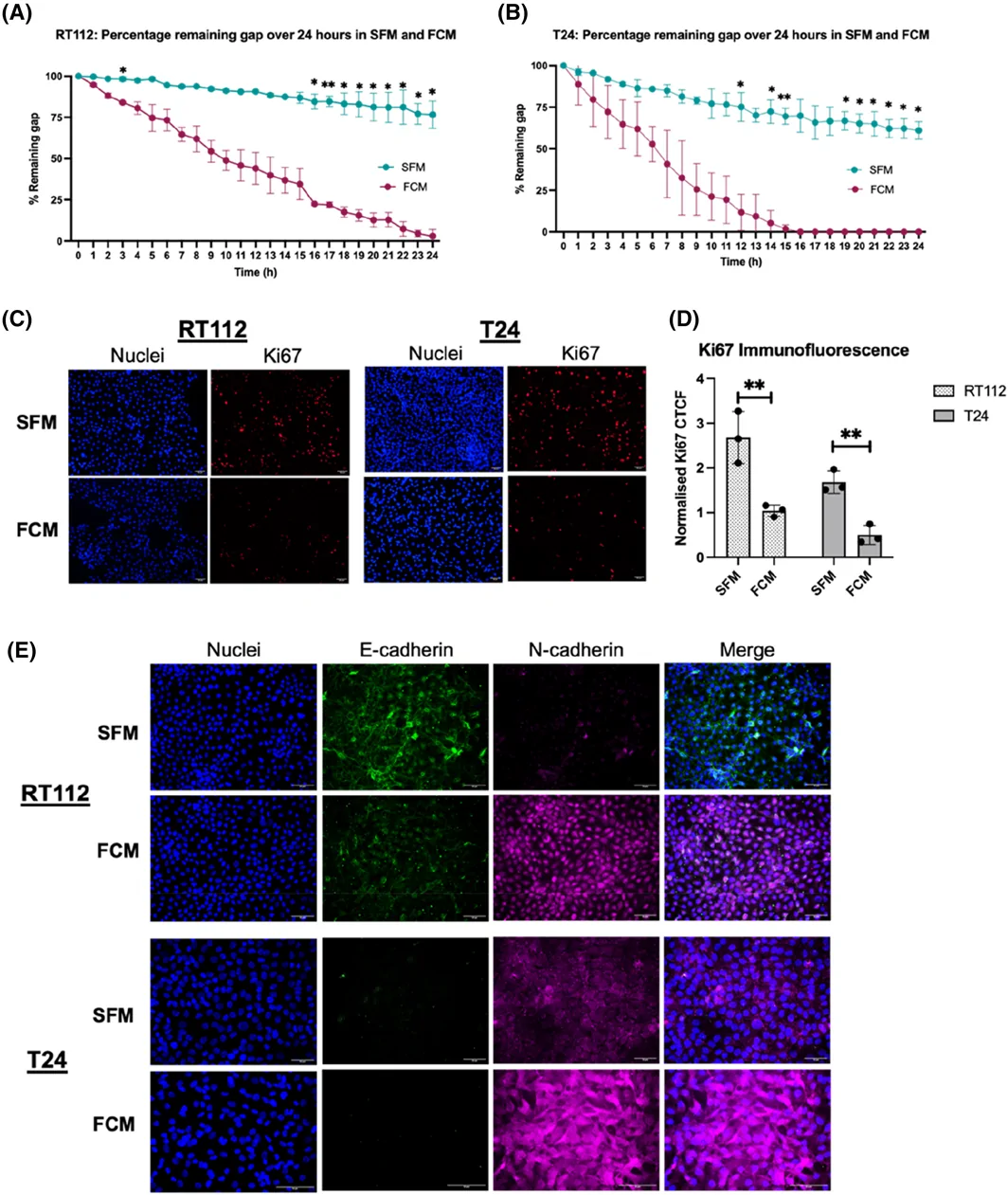
Effects of fibroblast‐conditioned medium on tumor cell proliferation, wound closure, and cadherin remodeling. (A, B) Wound‐healing assays showing the percentage of the initial wound gap remaining over 24 h in RT112 (A) and T24 (B) cells cultured in SFM or FCM. Data are presented as mean ± SD (*n* = 3). *p ≤ 0.05. (C) Representative Ki67 immunofluorescence images of RT112 and T24 cells cultured in SFM or FCM for 48 h. Scale bars = 50 μm. (D) Quantification of Ki67 fluorescence expressed as normalized CTCF, calculated as total Ki67 CTCF normalized to total DAPI CTCF. Data are presented as mean ± SD (*n* = 3). *p ≤ 0.05; **p ≤ 0.01. (E) Representative immunofluorescence images of E‐cadherin (green), N‐cadherin (magenta), and nuclei (DAPI, blue) following 48 h exposure to SFM or FCM. Scale bars = 50 μm. CTCF, corrected total cell fluorescence; DAPI, 4′,6‐diamidino‐2‐phenylindole; FCM, fibroblast‐conditioned medium; SFM, serum‐free medium.

We investigated the impact of fibroblast‐derived signals on bladder cancer cell behavior using FCM. To determine whether fibroblast‐derived soluble factors alter bladder cancer cell motility, RT112 and T24 cells were assessed using a culture‐insert wound‐healing assay. Removal of the insert generated a reproducible cell‐free gap, enabling serial quantification of gap closure over time (Supporting Information S1: Figure S1). In RT112 cells, FCM treatment consistently accelerated wound closure throughout the time course, producing a clear distinction between the two conditions. A similar effect was observed in T24 cells, where FCM also promoted faster wound closure, with treated cultures approaching near‐complete closure at later time points, while control cultures showed a more gradual decrease in wound area. At 24 h, FCM‐treated RT112 cells showed 97.1% wound closure compared with 23.4% in serum‐free controls. Similarly, FCM‐treated T24 cells reached 100% wound closure by 24 h, compared with 39.0% closure in serum‐free controls (Figure 3A,B). The combination of accelerated wound closure and reduced proliferative readouts was compatible with a shift toward a less proliferative, more motile phenotype.^16^

Consistent with the direction of the MTS findings, Ki67 immunofluorescence was significantly reduced after 48 h exposure to FCM in both RT112 and T24 cells. Quantification of total Ki67 fluorescence normalized to total 4′,6‐diamidino‐2‐phenylindole (DAPI) fluorescence showed a significant reduction in the relative proliferative signal in both cell lines compared with their corresponding SFM controls (Figure 3C,D). These findings provide an independent imaging‐based assessment supporting reduced tumor cell proliferation following exposure to fibroblast‐derived soluble factors.

Immunofluorescence after 48 h in FCM revealed a marked reduction in E‐cadherin and upregulation of N‐cadherin in both cell lines (Figure 3E). Together, these staining changes are consistent with EMT‐like cadherin remodeling.^17^

### 2.4 Bladder cancer cells induce fibroblast activation toward CAF‐like states

**FIGURE 4:**
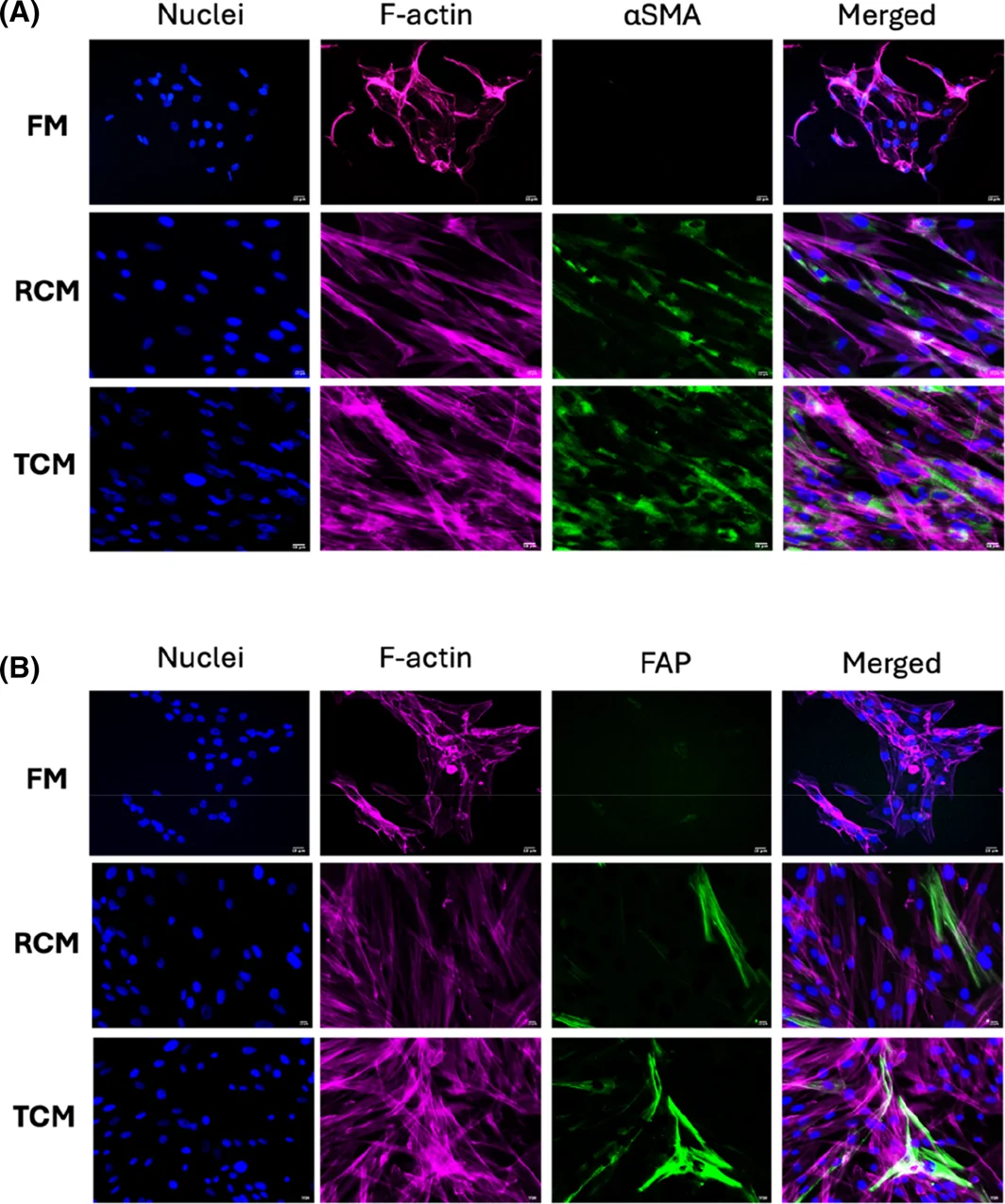
Cancer cell‐conditioned medium induces CAF‐like activation marker expression in normal fibroblasts. Immunofluorescence detection of αSMA (A) and FAP (B) in NFs after 48 h of exposure to cancer cell‐conditioned medium. Green fluorescence indicates positive staining. Images taken at 20× magnification. Scale bars: 10 μm. αSMA, α‐smooth muscle actin; CAF, cancer‐associated fibroblast; FAP, fibroblast activation protein; NFs, normal fibroblasts.

To investigate whether cancer cells influence fibroblast activation, we first examined the effect of cancer cell‐conditioned medium on NFs. Specifically, we assessed the induction of CAF‐like features by evaluating the expression of CAF‐associated activation markers, α‐smooth muscle actin (αSMA) and fibroblast activation protein (FAP), after 48 h of treatment. As shown in Figure 4A,B, exposure of NFs to RCM or TCM for 48 h increased αSMA and FAP staining by immunofluorescence, particularly in TCM‐treated cells. To extend the assessment beyond αSMA and FAP, we also examined PDGFRβ expression following exposure to cancer cell‐conditioned medium. TCM significantly increased PDGFRβ fluorescence, whereas RCM produced a non‐significant upward trend (Supporting Information S1: Figure S2). These data provide additional marker‐level support for rapid fibroblast phenotypic activation.

**FIGURE 5:**
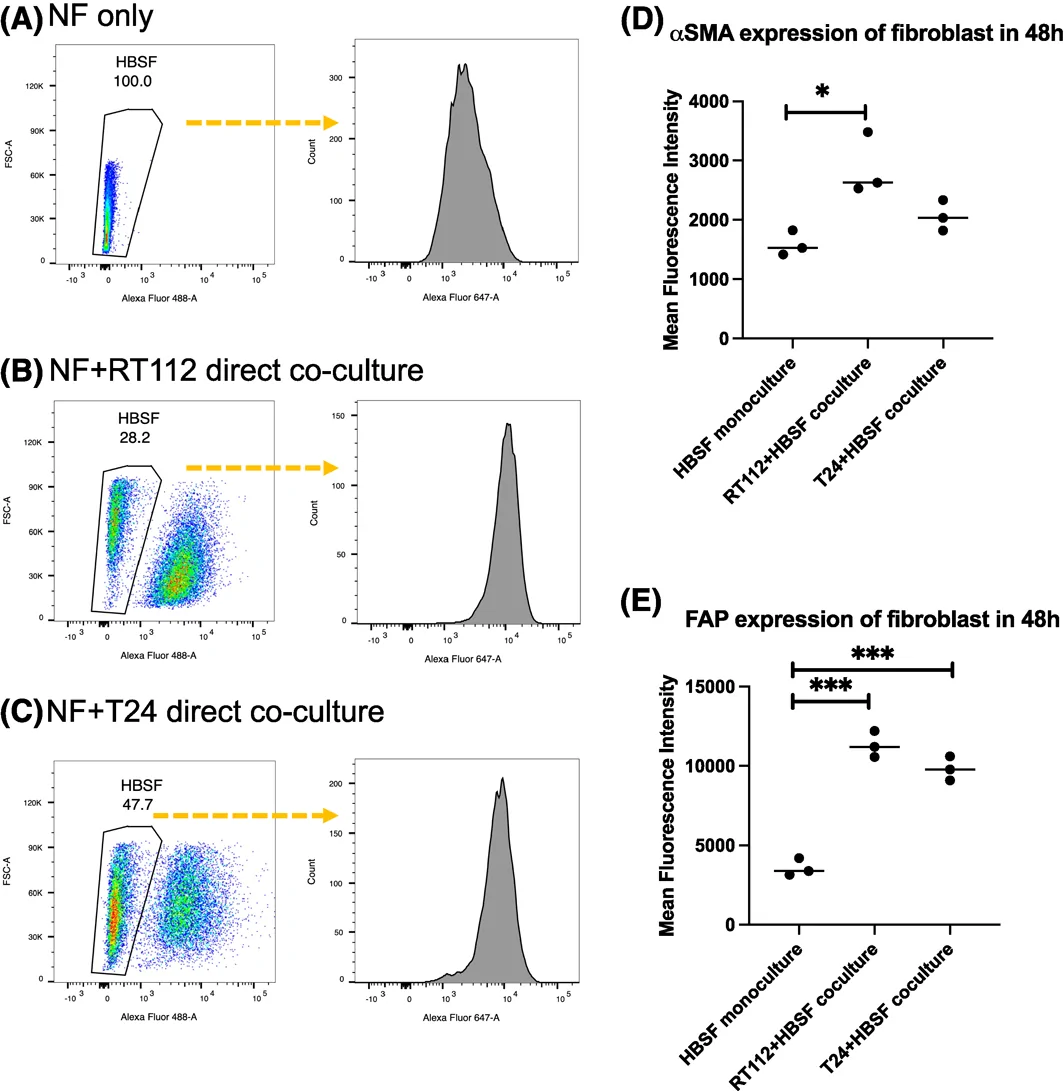
Direct co‐culture with bladder cancer cells upregulates CAF‐like activation markers in normal fibroblasts. (A–C) Representative flow cytometry gating for NFs in monoculture (A), co‐culture with RT112 (B), and co‐culture with T24 (C) after 48 h. Gates identify NFs as CellTracker Green‐negative (Alexa Fluor 488‐). Histograms show αSMA and FAP staining (Alexa Fluor 647) in gated NFs. (D, E) MFI for αSMA (D) and FAP (E) in NF monoculture versus co‐culture with RT112 or T24. Data are presented as mean ± SD (*n* = 3). *p ≤ 0.05; ***p ≤ 0.001. αSMA, α‐smooth muscle actin; CAF, cancer‐associated fibroblast; FAP, fibroblast activation protein; MFI, mean fluorescence intensity; NFs, normal fibroblasts.

To quantify this in a direct contact model, NFs were co‐cultured with RT112 or T24, and activation markers were measured by flow cytometry, gating specifically on the NF population (Figure 5A–C). Basal αSMA and FAP levels were low in NF monoculture (1590.7 ± 210.4 and 3575.0 ± 552.2 mean fluorescence intensity [MFI], respectively). Co‐culture with RT112 drove αSMA to 2880.0 ± 522.7 MFI and FAP to 11,325.0 ± 823.0 MFI, a greater than three‐fold induction for FAP, while T24 co‐culture yielded comparable FAP induction (9823.3 ± 755.5 MFI) but more modest αSMA upregulation (2063.7 ± 257.6 MFI; Figure 5D,E). The differing αSMA responses between RT112 and T24, despite broadly comparable FAP induction, indicate cell‐line‐dependent differences in fibroblast activation‐marker profiles.

Spatial organization of tumor‐stromal interactions, examined in 2D and 3D co‐culture models, is shown in Supporting Information S1: Figure S3. In 2D co‐culture, PanCK‐positive tumor cells maintained discrete epithelial clusters while vimentin‐positive fibroblasts adopted elongated morphologies and extended processes into surrounding tumor regions, forming a structured interface not observed in monoculture. T24 cells exhibited low‐level vimentin expression consistent with their partial mesenchymal character. In 3D co‐culture spheroids, fibroblasts were distributed throughout the spheroid mass rather than segregated peripherally, indicating close spatial integration between tumor and stromal compartments in both RT112 and T24 models. These spatial findings support the paracrine and contact‐mediated crosstalk characterized in the preceding experiments.

### 2.5 Cancer‐fibroblast crosstalk attenuates MMC efficacy in a ratio‐dependent manner

**FIGURE 6:**
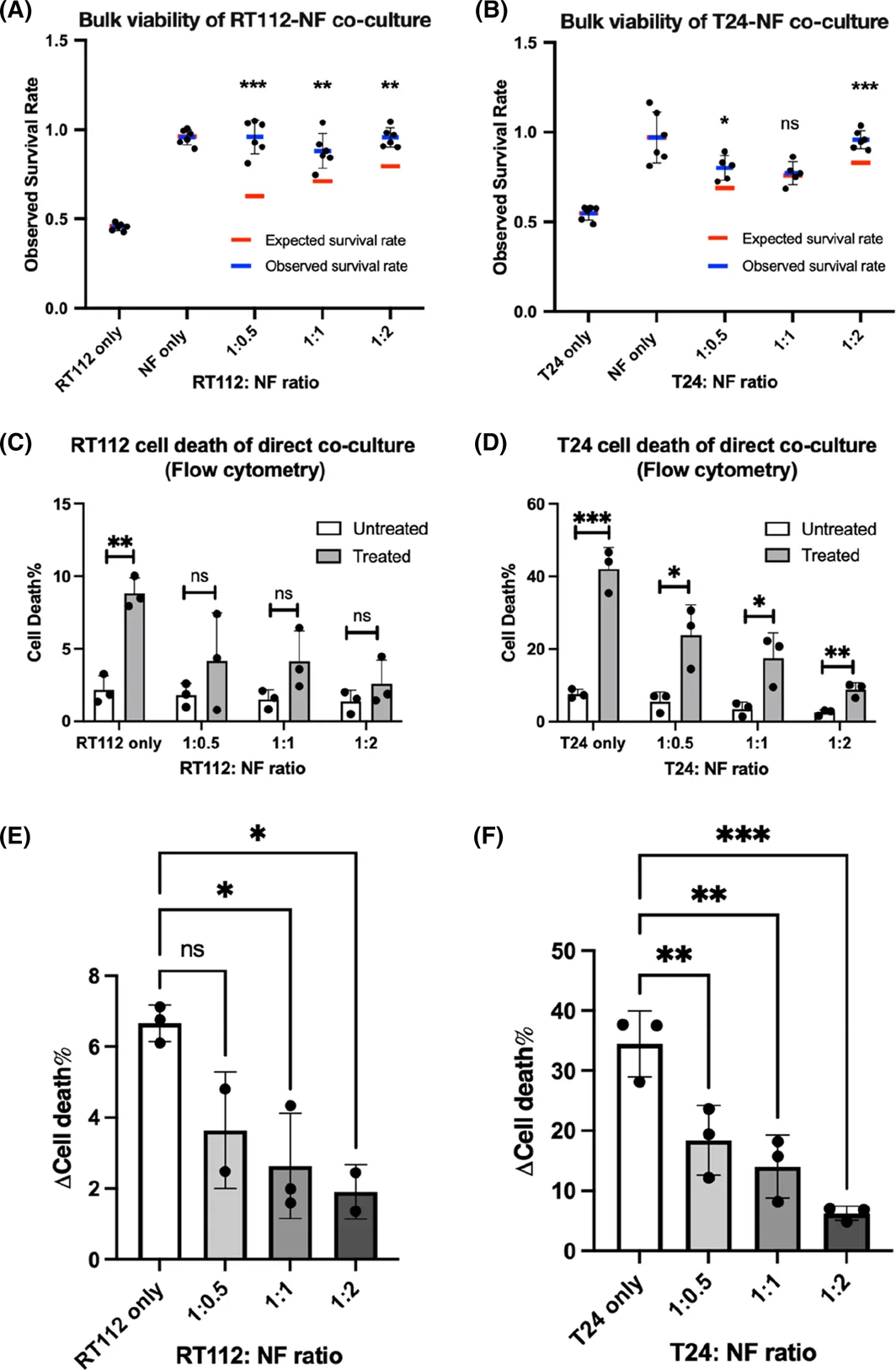
Fibroblast co‐culture attenuates mitomycin C cytotoxicity in bladder cancer cells in a ratio‐dependent manner. (A, B) MTS assay of RT112 (A) and T24 (B) cells treated with MMC (20 μM, 1 h) in direct co‐culture with NFs at increasing cancer‐to‐fibroblast ratios. Observed survival is compared with expected survival from monoculture responses, assuming no interaction between populations. Data are mean ± SD; *n* = 6 independent experiments. (C, D) Flow cytometry‐based cell death quantification in CellTracker Red‐labeled cancer cells co‐cultured with NFs, for RT112 (C) and T24 (D). (E, F) ΔCell death (%), calculated as dead cell fraction in MMC‐treated minus untreated condition per ratio, for RT112 (E) and T24 (F). Data are mean ± SD; *n* = 3 independent experiments. *p ≤ 0.05; **p ≤ 0.01; ***p ≤ 0.001. MMC, mitomycin C; MTS, 3‐(4,5‐dimethylthiazol‐2‐yl)‐5‐(3‐carboxymethoxyphenyl)‐2‐(4‐sulfophenyl)‐2H‐tetrazolium; NFs, normal fibroblasts.

To determine whether stromal abundance modulates chemotherapy response, RT112 and T24 cells were treated with MMC (20 μM, 1 h) in direct co‐culture with NFs at increasing cancer‐to‐fibroblast ratios. Bulk viability was assessed by MTS assay, comparing observed co‐culture survival against an expected value calculated from monoculture responses, assuming no interaction between populations. In RT112‐NF co‐culture, observed survival significantly exceeded the expected values across all fibroblast ratios (Figure 6A). In T24‐NF co‐culture, observed survival rates of 1:0.5 and 1:2 ratio co‐culture were significantly higher than expected survival rates (Figure 6B). As bulk assays cannot distinguish whether this increase reflects true protection of cancer cells or simply the contribution of a less sensitive fibroblast population, we next performed flow cytometry to quantify cell death specifically within the cancer cell compartment. By separating CellTracker Red‐labeled cancer cells from NFs and gating the dead cell population using the Zombie NIR viability kit, we found that the percentage of cell death induced by MMC in cancer cells was reduced in co‐culture conditions compared with monoculture controls in both RT112 and T24 (Figure 6C,D). To further isolate the treatment‐specific effect, we calculated ΔCell death% as the difference between treated and untreated within each ratio condition. This showed that the net cytotoxic effect of MMC on cancer cells was progressively reduced in the presence of NFs (Figure 6E,F), supporting that fibroblast co‐culture attenuates drug‐induced killing rather than simply altering baseline viability. Together, these findings show that fibroblast co‐culture attenuates MMC‐induced cancer cell killing in vitro.

## 3 DISCUSSION

Tumor‐stroma crosstalk is a recognized driver of cancer progression and therapy resistance, yet the specific role of NFs, an important stromal population encountered by invading bladder cancer cells, has remained largely uncharacterized. This study identifies rapid NF‐tumor crosstalk as a bidirectional and functionally important process in bladder cancer. The early‐stage NMIBC transcriptomic analysis provides clinical context for the experimental findings. Fibroblast‐associated scores were higher in T1 than Ta disease and enriched in EORTC high‐risk tumors, suggesting that fibroblast‐related programs are linked to both anatomical invasion and established clinical risk. Although fibroblast‐high tumors showed poorer PFS in the unadjusted analysis, this association was attenuated after adjustment for EORTC risk group and should not be interpreted as providing independent prognostic information. Experimentally, NF‐derived soluble factors reduced bladder cancer cell proliferation while enhancing wound closure and EMT‐like cadherin remodeling. Reciprocally, direct tumor co‐culture rapidly activated NFs toward CAF‐like states within 48 h, with increased αSMA and FAP expression. Functionally, increasing fibroblast abundance progressively attenuated MMC‐induced cancer‐cell killing. Together, these data support a model in which NFs are not passive bystanders in the bladder tumor microenvironment but are rapidly recruited into a tumor‐promoting and therapy‐modifying stromal dialog.

A major strength of this study is the focus on NFs rather than established CAFs. CAFs are widely recognized as drivers of tumor progression and treatment resistance, but this has left an important gap in understanding how CAF‐like states emerge during early disease. In bladder cancer, this question is particularly relevant because tumor cells invading into the lamina propria encounter resident NFs early in progression. Our findings suggest that this first stromal encounter is associated with reciprocal phenotypic changes: fibroblast‐derived signals promote enhanced wound closure and EMT‐like cadherin remodeling in tumor cells, while tumor cells rapidly induce CAF‐like fibroblast activation features within 48 h. These findings support a working model linking early fibroblast‐tumor contact, stromal activation and progression‐associated tumor plasticity.

The observation that FCM reduced proliferation but accelerated wound closure is compatible with a less proliferative, more motile phenotype. Rather than uniformly increasing tumor growth, fibroblast‐derived cues appear to shift bladder cancer cells toward a more motile phenotype. This distinction is important because aggressive behavior in early bladder cancer may not always be reflected by increased proliferation alone. A tumor cell population that proliferates less but displays greater motility may be better positioned for local invasion and progression. Future studies incorporating transwell migration or matrix‐invasion assays will be needed to provide density‐independent confirmation of whether fibroblast‐derived signals enhance migration or matrix invasion. The accompanying EMT‐like cadherin remodeling strengthens this interpretation and aligns with the patient‐level finding that fibroblast‐associated scores correlate positively with EMT scores. However, because EMT transcription factors and invasion assays were not assessed, these findings should be interpreted as partial EMT‐associated plasticity rather than complete EMT.

The reciprocal fibroblast activation observed within 48 h indicates that NFs can acquire CAF‐like features rapidly after tumor exposure. Increased αSMA suggests myofibroblast‐like activation, while increased FAP indicates acquisition of a stromal state frequently associated with tissue remodeling, invasion, and poor outcome.^18^ Importantly, these findings suggest that CAF‐like activation should not be considered only a late or stable endpoint. Instead, fibroblast activation may be a dynamic and early response to tumor contact.

The differential αSMA and FAP responses observed in direct RT112 and T24 co‐cultures, together with the differing PDGFRβ responses following exposure to RCM and TCM, are consistent with cell‐line‐dependent heterogeneity in early CAF‐like activation features.^19^ Although αSMA, FAP, and PDGFRβ provide marker‐level evidence of fibroblast phenotypic activation, the present study did not assess broader transcriptional reprogramming, secretome alterations, matrix deposition, contractile function, or the long‐term stability of the induced phenotype. The findings should therefore be interpreted as rapid acquisition of CAF‐like activation features rather than evidence of complete or stable CAF conversion. Candidate pathways such as TGFβ, IL‐6, HGF, CXCL12, STAT3, and ECM‐associated signaling may contribute to these reciprocal interactions, but these mediators were not directly tested in the present study. Future experiments using cytokine profiling, pathway inhibition, neutralizing antibodies, and phospho‐signaling assays will be required to identify the specific tumor‐ and fibroblast‐derived signals responsible for the observed phenotypic changes.

The MMC response data provide the important functional consequence of this crosstalk. Fibroblast‐containing co‐cultures were less sensitive to MMC, and the attenuation of MMC‐induced cytotoxicity became greater with increasing fibroblast abundance. This ratio‐dependent effect is biologically relevant in vitro and may have potential clinical implications. In NMIBC, particularly after resection, residual tumor cells may be exposed to MMC within a complex stromal and wound‐associated microenvironment rather than in isolation. A fibroblast‐rich or rapidly activated stromal compartment could therefore weaken the cytotoxic effect of MMC during the short intravesical exposure window. The use of cancer‐cell‐specific flow cytometry and ΔCell death analysis supports that this reflects reduced MMC‐induced killing within the tumor cell compartment rather than only altered bulk co‐culture viability. Nevertheless, the mechanism underlying this reduced MMC sensitivity remains unresolved. Altered proliferative state or cell‐cycle distribution may contribute, alongside other plausible mechanisms such as paracrine survival signaling, contact‐mediated interactions, matrix‐associated effects, altered effective drug exposure, physical drug sequestration by fibroblasts, or changes in DNA damage response pathways. Future studies incorporating cell‐cycle analysis, DNA damage markers, MMC uptake or distribution assays, and pathway inhibition will be required to distinguish these possibilities.

Several additional experimental limitations warrant mention. The fibroblast‐associated scores in this study were derived from bulk transcriptomic data and therefore cannot assign gene expression to fibroblasts with single‐cell resolution. These scores should therefore be interpreted as fibroblast‐associated transcriptional programs rather than direct measurements of fibroblast abundance. Future validation using single‐cell, spatial transcriptomic approaches will be required to define the precise cellular sources and functional heterogeneity of these stromal programs. The experimental work used one commercially sourced normal bladder stromal fibroblast preparation, two established bladder cancer cell lines, and short‐term conditioned‐medium or co‐culture systems. These models allowed controlled testing of reciprocal fibroblast‐tumor interactions, but they do not capture the full heterogeneity of patient‐derived CAF states, tumor genotypes, immune context, or extracellular matrix organization. In particular, CAF activation is likely to be patient‐specific and influenced by tissue architecture, chronic tumor exposure, and additional stromal or immune cell populations that are not represented in the present reductionist models. Future work using multiple patient‐derived normal bladder fibroblast preparations, established CAFs, bladder cancer organoids, and ex vivo tissue models will be required to validate the generalizability of these findings and determine whether similar early fibroblast activation states occur in patient tumors.

In conclusion, this study provides evidence that NF‐tumor crosstalk in bladder cancer is rapid, bidirectional, and capable of modifying tumor‐cell phenotype and MMC response in vitro. NF‐derived signals can promote EMT‐like cadherin remodeling and enhanced wound closure; bladder cancer cells can rapidly induce CAF‐like fibroblast activation, and the resulting stromal interaction reduces MMC sensitivity in a ratio‐dependent manner. These findings identify NFs as active early participants in bladder tumor‐stromal interactions and support consideration of stromal context when evaluating intravesical therapy for NMIBC.

## 4 MATERIALS AND METHODS

### 4.1 Transcriptomic analysis of an early‐stage NMIBC cohort

Publicly available transcriptomic and clinical annotation data from an early‐stage non‐muscle‐invasive bladder cancer (NMIBC) cohort were analyzed to assess the clinical relevance of fibroblast‐associated transcriptional programs. The transcriptomic dataset analyzed in this study is publicly available from ArrayExpress under accession E‐MTAB‐4321. The original sequencing data are available through the European Genome‐phenome Archive under accession EGAS00001001236, as described by Hedegaard et al.^20^ Gene expression data and corresponding sample metadata were downloaded from the E‐MTAB‐4321 cohort, which contains transcriptomic profiles from NMIBC tumors with available clinicopathological annotation, including tumor stage, grade, EORTC risk classification, and PFS information. Samples with missing key clinical variables or survival information were excluded from the corresponding analyses.

Expression data were processed in R. Gene expression values were log2‐transformed where required and matched to clinical metadata using sample identifiers. A fibroblast‐associated score was calculated for each tumor using a predefined panel of fibroblast and stromal activation‐associated genes: FAP, ACTA2, TAGLN, PDGFRB, COL1A1, COL1A2, FN1, POSTN, THY1, CXCL12, and TGFB1. For each gene, expression values were *z*‐score normalized across all samples, and the fibroblast‐associated score for each sample was calculated as the mean of the available *z*‐scored genes. Samples were then stratified into fibroblast‐low and fibroblast‐high groups using the median fibroblast‐associated score as the cut‐off.

This panel was selected to capture a fibroblast/stromal activation‐associated program rather than a fibroblast‐exclusive signature. It includes canonical fibroblast and CAF‐associated markers, extracellular matrix genes, and stromal signaling components. Because several genes in the panel may also be expressed by other stromal or tumor‐adjacent cell populations in bulk tumor data, the score was interpreted as a stromal/fibroblast‐associated transcriptional program rather than a cell‐type‐specific measure of fibroblast abundance. For comparison with an external published fibroblast marker set, fibroblast transcriptomic markers from MCP‐counter were used to derive sample‐level scores. For each available gene, expression values were *z*‐score normalized across tumors, and scores were calculated as the mean of the available *z*‐scored genes. This was performed using both the full MCP‐counter fibroblast marker set and the MCP‐counter restricted fibroblast marker subset, defined as genes annotated as restricted transcriptomic markers in the MCP‐counter supplementary table rather than by excluding genes overlapping with the predefined score. Associations with the predefined fibroblast/stromal‐associated score were assessed using Spearman rank correlation.

To assess EMT‐associated transcriptional features, an EMT score was calculated as the difference between the mean *z*‐scored expression of mesenchymal markers and the mean *z*‐scored expression of epithelial markers, adapted from previously described transcriptomic EMT scoring approaches.^21^ The mesenchymal score was calculated as the mean *z*‐scored expression of CDH2, VIM, SNAI1, SNAI2, ZEB1, ZEB2, and TWIST1, while the epithelial score was calculated using *z*‐scored CDH1 expression. The final EMT score was defined as the mesenchymal score minus the epithelial score. Correlation between fibroblast‐associated scores and EMT scores was assessed using Spearman's rank correlation.

Fibroblast‐associated scores were compared between Ta and T1 tumors, and between EORTC low‐risk and high‐risk groups, using Wilcoxon rank‐sum tests. To investigate resistance‐associated transcriptional features, mRNA expression levels of STAT3, AXL, BCL2L1, and MCL1 were compared between fibroblast‐low and fibroblast‐high tumors using Wilcoxon rank‐sum tests.

PFS was analyzed using available clinical follow‐up data. PFS time was defined according to the survival annotation provided in the cohort metadata, and progression events were coded using the corresponding progression indicator. Kaplan–Meier curves were generated to compare PFS between fibroblast‐low and fibroblast‐high groups, and differences were assessed using the log‐rank test.

All statistical analyses and data visualization were performed in R using standard packages including dplyr, ggplot2, survival, and survminer. A two‐sided *p*‐value <0.05 was considered statistically significant.

### 4.2 Cell culture

Bladder cancer cell lines RT112 and T24, together with human bladder stromal fibroblasts (HBSF; referred to as NFs), were used in this study. RT112 cells (official cell line name: RT‐112; RRID: CVCL_1670; *Homo sapiens*; female; urinary bladder carcinoma) were kindly provided by Dr. Mark Linch's laboratory at the UCL Cancer Institute in October 2019. T24 cells (official cell line name: T24; RRID: CVCL_0554; *H. sapiens*; female; urinary bladder carcinoma) were obtained from the American Type Culture Collection in January 2021. HBSF/HBdSF (ScienCell Research Laboratories, #4330; *H. sapiens*; sex not specified by supplier; normal bladder stromal tissue; no RRID available for this primary fibroblast preparation) were obtained from ScienCell Research Laboratories in July 2022. RT112 and T24 cells were selected as widely used and well‐characterized bladder cancer cell line models representing distinct bladder cancer phenotypes, while HBSF were used as a normal stromal fibroblast model relevant to the bladder microenvironment. Cell line identity was confirmed based on authenticated source/laboratory stocks and supplier documentation, and none of the cell lines used in this study have been reported as misidentified or cross‐contaminated at the time of manuscript preparation. All cell lines were confirmed to be free of mycoplasma contamination during the experimental period using the Lonza MycoAlert Mycoplasma Detection Kit. Cancer cells were cultured in DMEM (Gibco) with 10% FBS (Fisher Scientific) and 1% Penicillin/Streptomycin (Fisher Scientific). NFs were maintained in fibroblast medium (FM; ScienCell #2301). All cells were maintained at 37°C, 5% CO_2_, and passaged at 80%–90% confluence using Accutase (Merck).

### 4.3 Conditioned medium preparation

Serum‐free medium was used to eliminate FBS‐associated variability. RT112, T24, and NFs were seeded separately in T150 flasks. After 24 h, medium was replaced with DMEM or FM containing 1% Penicillin/Streptomycin. After 48 h of incubation, the conditioned media (RCM, TCM, and FCM) were harvested, centrifuged to remove debris, filtered through 0.2 μm filters (Fisher Scientific), and stored at 4°C for subsequent experiments.

### 4.4 MTS cell proliferation assay

For conditioned medium experiments, 3000 cancer cells or NFs per well were seeded in 96‐well plates, treated with conditioned or serum‐free medium after 24 h, and assessed by MTS assay (Promega) at 48 and 96 h. For MMC experiments, RT112 or T24 cells were co‐cultured with NFs at 1:0.5, 1:1, and 1:2 ratios (6000 total cells/well). After 24 h, cells were treated with MMC (Selleck Chemicals, 20 μM, 1 h), washed with PBS (Fisher Scientific), and incubated in fresh medium for 72 h before the MTS assay. To determine whether co‐culture survival exceeded what would be expected from each cell population independently, an expected survival value was calculated for each ratio as a weighted average of monoculture MMC responses: Expected survival (%) = [f_cancer × Survival_cancer] + [f_NF × Survival_NF], where f_cancer and f_NF are the fractional contributions of each population at the given seeding ratio, and Survival_cancer and Survival_NF are the MTS‐measured survival percentages of cancer cells and NFs in MMC‐treated monoculture, respectively.

### 4.5 Wound healing assay

RT112 (2.5 × 10^5^) and T24 (2.0 × 10^5^) cells were seeded to confluence in 24‐well plates. A wound gap was created using Ibidi Culture‐Insert 2‐Well inserts, which were removed after 24 h. Medium was replaced with either SFM or FCM at the time of insert removal (*t* = 0). Images were captured hourly for 24 h using a Leica MICA microscope and residual wound gap (%) was measured using LAS X software, normalized to the *t* = 0 gap width.

### 4.6 Immunofluorescence

For indirect co‐culture, NFs or cancer cells (3 × 10^4^/well) were seeded on Lab‐Tek 8‐well chamber slides, treated with conditioned medium for 48 h, then fixed in 4% formaldehyde (FA), permeabilized with 0.2% Triton X‐100 (Fisher Scientific), and blocked with 5% normal goat serum/1% BSA. NFs were stained with anti‐FAP (rabbit monoclonal, Cell Signaling Technology, CST, #66562) and anti‐αSMA (rabbit monoclonal, CST, #19245) at 1:100, followed by anti‐rabbit Alexa Fluor 488 and Alexa Fluor 555 Phalloidin (Invitrogen). For PDGFRβ immunofluorescence, NFs were stained with anti‐PDGFRβ (rabbit monoclonal, CST, #3169) at 1:100, followed by anti‐rabbit Alexa Fluor 633 secondary antibody (Invitrogen) at 1:500 and Alexa Fluor 555 Phalloidin (Invitrogen) to visualize F‐actin. Nuclei were counterstained with DAPI. Quantitative fluorescence images were acquired using identical exposure, gain, and field dimensions across treatment conditions within each experiment. Corrected total cell fluorescence was quantified in FIJI. Multiple imaging fields were averaged to generate one value for each independent biological experiment before statistical analysis.

Cancer cells were stained with anti‐E‐cadherin (mouse monoclonal, Invitrogen, #33‐4000; 10 μg/mL) and anti‐N‐cadherin (rabbit polyclonal, Invitrogen, #PA5‐19486; 1 μg/mL), followed by appropriate fluorescently labeled secondary antibodies. Nuclei were counterstained with DAPI (Fisher Scientific). For Ki67 immunofluorescence, RT112 and T24 cells were seeded on Lab‐Tek 8‐well chamber slides, treated with SFM or FCM for 48 h, then fixed in 4% FA, permeabilized with 0.2% Triton X‐100, and blocked with 5% normal goat serum/1% BSA. Cells were stained with a directly conjugated anti‐Ki67 antibody (rabbit recombinant monoclonal [SP6], Alexa Fluor 647, Abcam, ab281928) at 1:50, with nuclei counterstained with DAPI.

For 2D direct co‐culture and 3D spheroid immunofluorescence, RT112 or T24 cells and NFs were co‐seeded at a 1:1 ratio on 8‐well chamber slides (Lab‐Tek) and cultured for 48 h. Cells were fixed via 4% FA (Fisher Scientific) and stained for pan‐cytokeratin (mouse monoclonal, CST, #67306) and vimentin (rabbit monoclonal, CST, #5741) at 1:100, with DAPI counterstain. For 3D spheroid generation, RT112 or T24 cells and NFs were mixed at a 1:1 ratio and seeded in ultra‐low attachment 96‐well round‐bottom plates (Fisher Scientific) at a total density of 3000 cells per well. Spheroids were allowed to form over 72 h and imaged by Leica SP8 confocal microscopy following fixation and immunofluorescence staining for PanCK and vimentin at 1 in 100 dilution as well.

### 4.7 Flow cytometry

Prior to co‐culture, RT112 or T24 cancer cells were labeled with CellTracker Green CMFDA (Thermo Fisher Scientific, C7025; 10 μM in SFM for 30 min at 37°C) to enable downstream discrimination from NFs by flow cytometry. Labeled cancer cells were then co‐seeded with NFs in T75 flasks at a 1:1 ratio for 48 h. Cells were detached with Accutase and fixed with 4% FA. After permeabilization with 0.2% Triton X‐100 and blocking with 5% normal goat serum, cells were stained with αSMA or FAP primary antibodies (1:100) for 45 min. Secondary antibody (anti‐rabbit Alexa Fluor 647) was applied for 30 min at 1:500. After washing, cells were resuspended in 1% BSA and analyzed using a BD LSR Fortessa flow cytometer. Data were processed using FlowJo software.

To enable population‐specific cell death analysis, cancer cells (RT112 or T24) were labeled with CellTracker Red CMTPX (Thermo Fisher Scientific, C34552; 5 μM in DMEM for 30 min at 37°C) and NFs were labeled with CellTracker Green CMFDA (Thermo Fisher Scientific, C7025; 10 μM in DMEM for 30 min at 37°C) prior to co‐culture. Red‐labeled cancer cells were seeded with green‐labeled NFs at 1:0.5, 1:1, and 1:2 ratios in 6‐well plates (total 300,000 cells/well). After 24 h, cells were treated with MMC (20 μM, 1 h), washed with PBS, and incubated in fresh medium for 72 h. Cells were then detached with Accutase, pelleted, and resuspended in PBS. Cell viability was assessed using Zombie NIR Fixable Viability Kit (BioLegend, #423105; 1:1000 dilution in PBS for 15 min at room temperature, protected from light). After washing, cells were resuspended in 1% BSA/PBS and acquired on a BD LSRFortessa. Cancer cells were gated as CellTracker Red‐positive; Zombie NIR‐positive cells within this gate were scored as dead. Cell death% was defined as the percentage of Zombie NIR‐positive cells within the CellTracker Red‐positive population. ΔCell death% was calculated as: (cell death% in MMC‐treated condition) − (cell death% in matched untreated condition) for each co‐culture ratio, isolating the treatment‐specific cytotoxic effect from baseline viability differences. Data were processed in FlowJo. For all flow cytometry analyses, events were first gated using FSC‐A/SSC‐A to exclude debris, followed by singlet discrimination using FSC‐A/FSC‐H. For fibroblast activation assays, CellTracker Green‐positive cancer cells were excluded and CellTracker Green‐negative HBSF were analyzed for αSMA or FAP fluorescence intensity. For MMC cytotoxicity assays, CellTracker Red‐labeled cancer cells and CellTracker Green‐labeled HBSF were separated according to CellTracker fluorescence, and Zombie NIR‐positive events within the gated CellTracker Red‐positive cancer‐cell population were scored as dead cancer cells. Fluorescence thresholds were set using unstained, single‐stained, and secondary‐only controls, with compensation applied where required. Representative gating strategies for both assays are provided in Supporting Information S1: Figure S4.

### 4.8 Statistical analysis

In vitro data are presented as mean ± SD from three independent biological experiments unless otherwise stated. Where technical replicate wells or imaging fields were included within an experiment, these were averaged before statistical comparison so that the independent biological experiment was used as the unit of analysis. Comparisons between two independent groups were performed using unpaired two‐tailed *t*‐tests. Where observed values were compared with a single calculated expected value, one‐sample two‐tailed *t*‐tests were used. Comparisons involving more than two groups were performed using one‐way ANOVA followed by Dunnett's multiple‐comparisons test when treatment groups were compared with a single control. For transcriptomic cohort analyses, Wilcoxon rank‐sum tests, Spearman correlation, and Kaplan–Meier/log‐rank analysis were used as described above. A two‐sided *p*‐value <0.05 was considered statistically significant.

## AUTHOR CONTRIBUTIONS

**Jinhui Gao**: Conceptualization; methodology; investigation; funding acquisition; supervision; writing—original draft; writing—review and editing. **Cindy Xinyu Ji**: Investigation; methodology. **Ci Ren**: Investigation; methodology. **John Hines**: Writing—review and editing. **Eleanor Stride**: Funding acquisition; supervision; writing—review and editing. **Richard T. Bryan**: Funding acquisition; supervision; writing—review and editing. **Jennifer L. Rohn**: Funding acquisition; supervision; writing—review and editing.

## CONFLICT OF INTEREST STATEMENT

J. L. R. and E. S. have share options in AtoCap Ltd, whose focus is not related to the present manuscript. The remaining authors declare no conflicts of interest.

## ETHICS STATEMENT

This study used established human cell lines, a commercially sourced human bladder stromal fibroblast preparation, and publicly available de‐identified transcriptomic data. No new human participants or human tissue specimens were recruited or collected for this study, and no animal experiments were performed. Institutional ethics approval and informed consent were therefore not required.

## Supporting information

Supporting Information S1

## Data Availability

The transcriptomic dataset analyzed in this study is publicly available from ArrayExpress under accession E‐MTAB‐4321. The original sequencing data are available through the European Genome‐phenome Archive under accession EGAS00001001236. The remaining data supporting the findings of this study are available from the corresponding author upon reasonable request.
