# Supplementary material for: Rapid normal fibroblast‐tumor crosstalk promotes cancer‐associated fibroblast‐like activation and attenuates mitomycin C cytotoxicity in bladder cancer
Source: J Cell Commun Signal. 2026 Aug 24;20(3):e70110. doi: 10.1002/ccs3.70110 (PMC13501395; doi:10.1002/ccs3.70110)
Supplement: Supplementary file 1 [file CCS3-20-e70110-s001.docx]

# Supplementary Results

Supplementary Figure S1. Representative images showing accelerated wound closure following FCM exposure.

RT112 and T24 cells were seeded into two-well culture inserts to generate a defined cell-free gap. Following insert removal, cells were treated with SFM or FCM, and gap closure was monitored over time. Average changes in gap width were quantified as a measure of wound closure. Representative phase-contrast images are shown for RT112 and T24 cells at 0, 8, 16 and 24 h.

To assess whether soluble factors released by normal fibroblasts influence bladder cancer cell motility, RT112 and T24 cells were exposed to FCM in a culture-insert wound-healing assay. Removal of the insert generated a reproducible cell-free gap, enabling temporal assessment of wound closure by measuring changes in gap width over time. Representative images showed that fibroblast-conditioned medium accelerated wound closure in both RT112 and T24 cells compared with serum-free controls, with treated cultures displaying visibly reduced gap widths by 8-16 h and near-complete closure at later time points. This effect was particularly evident in the fibroblast-conditioned medium groups, which closed the gap more rapidly than their matched serum-free controls. These observations support the quantitative wound-healing analysis shown in the main figure and indicate that fibroblast-derived soluble factors accelerate wound closure, consistent with a more motile, stromal-influenced phenotype.

Supplementary Figure S2. Cancer cell-conditioned medium alters PDGFRβ expression in normal bladder stromal fibroblasts.

(a) Representative immunofluorescence images of normal bladder stromal fibroblasts cultured for 48 h in fibroblast medium control (FM), RT112-conditioned medium (RCM) or T24-conditioned medium (TCM). Cells were stained for PDGFRβ (red), F-actin/phalloidin (magenta) and nuclei/DAPI (blue). Merged images are shown. Scale bars = 50 μm. (b) Quantification of PDGFRβ corrected total cell fluorescence (CTCF) from immunofluorescence images. Individual biological replicates and the mean are shown; n = 3. **p ≤ 0.01; ns, not significant.

To extend the assessment of fibroblast activation beyond αSMA and FAP, PDGFRβ was assessed by immunofluorescence in normal bladder stromal fibroblasts following 48 h exposure to cancer cell-conditioned medium. Compared with fibroblast medium control, T24-conditioned medium significantly increased PDGFRβ fluorescence, while RT112-conditioned medium showed a non-significant upward trend. These findings provide additional supporting evidence that tumour-derived soluble factors induce early CAF-like activation features in normal bladder fibroblasts. However, because PDGFRβ was assessed as a single additional marker and CAF identity is not defined by marker expression alone, these data are interpreted as supportive rather than definitive evidence of CAF conversion.

Supplementary Figure S3: Spatial organisation of bladder cancer cells and normal fibroblasts in 2D and 3D direct co-culture. PanCK (red) marks epithelial cells, vimentin (green) marks stromal fibroblasts, and nuclei are stained with DAPI (blue). Scale bars: 50 µm.

Tumour-fibroblast spatial organisation was examined using direct co-culture of bladder cancer cells with NFs, followed by immunofluorescence staining for epithelial (PanCK) and mesenchymal (vimentin) markers. In direct 2D co-culture, tumour and stromal cells formed a structured interface. PanCK-positive tumour cells maintained epithelial clusters, whereas vimentin-positive fibroblasts adopted elongated morphologies and extended processes surrounding tumour cell regions, creating an organised tumour-stromal architecture not observed in monoculture conditions. Additionally, T24 cells showed low-level vimentin staining consistent with the mesenchymal-associated phenotype of this cell line.

To further investigate tumour-stromal organisation in a three-dimensional context, tumour-fibroblast spheroids (co-culture at 1:1 ratio) were generated and analysed by Leica SP8 confocal microscopy. In these spheroids, PanCK-positive tumour cells formed the main spheroid structure, while vimentin-positive fibroblasts were distributed throughout the spheroid mass, indicating close spatial association between tumour and stromal components. Similar organisational patterns were observed in both RT112 and T24 co-culture spheroids.

Supplementary Figure S4. Representative flow cytometry gating strategies for fibroblast activation and cancer-cell-specific MMC cytotoxicity assays.

(a-d) Representative gating workflow for fibroblast activation assays. Events were first gated by FSC-A/SSC-A to exclude debris (a), followed by singlet discrimination using FSC-A/FSC-H (b). In direct co-cultures containing CellTracker Green-labelled cancer cells and unlabelled HBSF, CellTracker Green-positive cancer cells were excluded and CellTracker Green-negative HBSF were gated for downstream analysis (c). αSMA or FAP fluorescence intensity was then quantified within the gated HBSF population (d).

(e-h) Representative gating workflow for MMC cytotoxicity assays. Events were first gated by FSC-A/SSC-A to exclude debris (e), followed by singlet discrimination using FSC-A/FSC-H (f). In direct co-cultures containing CellTracker Red-labelled cancer cells and CellTracker Green-labelled HBSF, the two cell populations were separated according to CellTracker fluorescence, with cancer cells identified as CellTracker Red-positive and HBSF identified as CellTracker Green-positive (g). Zombie NIR staining was then assessed within the gated cancer-cell population, and Zombie NIR-positive events were quantified as dead cancer cells (h). Fluorescence thresholds were set using unstained, single-stained and secondary-only controls, with compensation applied where required.

Representative gating workflows were included to clarify the population-specific flow cytometry analyses used in this study. For fibroblast activation assays, debris and doublets were excluded before CellTracker Green-positive cancer cells were removed from the analysis. The remaining CellTracker Green-negative HBSF population was used for quantification of αSMA or FAP fluorescence intensity, allowing fibroblast activation marker expression to be assessed specifically within the stromal compartment.

For MMC cytotoxicity assays, debris and doublets were similarly excluded before CellTracker fluorescence was used to separate CellTracker Red-labelled cancer cells from CellTracker Green-labelled HBSF. Cell death was then quantified specifically within the gated cancer-cell population using Zombie NIR positivity. These gating strategies enabled separate analysis of fibroblast activation and tumour-cell viability within direct co-culture systems.

Supplementary Figure S5. Correlation between the predefined fibroblast/stromal-associated score and the published MCP-counter fibroblast marker set in the UROMOL NMIBC cohort. (a) Correlation between the predefined fibroblast/stromal-associated score and a score derived from the published MCP-counter fibroblast marker set. (b) Correlation between the predefined fibroblast/stromal-associated score and a score derived from the restricted MCP-counter fibroblast marker set. Each point represents one tumour. Signature scores were calculated using mean z-scored gene expression values. Associations were assessed using Spearman rank correlation.

To assess whether the predefined fibroblast/stromal-associated score was concordant with an independently published fibroblast marker set, we compared it with the MCP-counter fibroblast transcriptomic markers in the same UROMOL NMIBC cohort. The predefined score showed a strong positive correlation with the published MCP-counter fibroblast marker-set score (Spearman rho = 0.897, p < 2.2e-16, n = 476), with a similarly strong association observed using the restricted MCP-counter fibroblast marker set (rho = 0.900, p < 2.2e-16, n = 476; Supplementary Figure S5). These findings support the convergent validity of the predefined fibroblast/stromal-associated score, while reinforcing that it should be interpreted as a bulk stromal/fibroblast-associated transcriptional programme rather than a fibroblast-specific abundance measure. The full MCP-counter fibroblast marker set comprised PLXNA3, CEMIP, FGF7, KGFLP1, KGFLP2, PRR16, C1S, TAGLN, MTSS1L, LRRN4CL, MYL9, COL3A1, DCN, PDGFRB, COL1A1, FBLN1, FBLN5, PDGFRA, IGFBP5, STC2, CNN1, LPAR1, PPP1R3C, LOX, TNFRSF11B, THY1, COL6A2, EFEMP2, MFGE8, C1R, COL6A1, ELN, ANGPTL2, CREB3L1, SVEP1, MXRA8, PAMR1, PARVA, ADAMTS2, HSPB6, LOC100287387, GREM1, HSPB7, CD248, COPZ2, PRRX2, VGLL3, GREM2, GLT8D2, ITGA11, CCDC80, VASN, PCDH18, PODN, FIBIN, LINC01279, TMEM119, RGMB, PLAC9, SCARF2, WISP1, CNTN3, COL12A1, MASP1, KRTAP1-5 and LOC100507165. The restricted MCP-counter fibroblast marker subset comprised TAGLN, COL3A1, DCN, COL1A1, COL6A2, COL6A1, PAMR1 and GREM1. Genes overlapping with the predefined fibroblast/stromal-associated score were TAGLN, PDGFRB, COL1A1 and THY1 for the full set, and TAGLN and COL1A1 for the restricted subset.
